# LiDAR-Based Glass Detection for Improved Occupancy Grid Mapping

**DOI:** 10.3390/s21072263

**Published:** 2021-03-24

**Authors:** Haileleol Tibebu, Jamie Roche, Varuna De Silva, Ahmet Kondoz

**Affiliations:** Institute of Digital Technologies, Loughborough University London, 3 Lesney Avenue, London E20 3BS, UK; A.J.Roche@lboro.ac.uk (J.R.); V.D.De-Silva@lboro.ac.uk (V.D.S.); A.Kondoz@lboro.ac.uk (A.K.)

**Keywords:** glass detection, occupancy grid mapping, LiDAR noise reduction, localisation

## Abstract

Creating an accurate awareness of the environment using laser scanners is a major challenge in robotics and auto industries. LiDAR (light detection and ranging) is a powerful laser scanner that provides a detailed map of the environment. However, efficient and accurate mapping of the environment is yet to be obtained, as most modern environments contain glass, which is invisible to LiDAR. In this paper, a method to effectively detect and localise glass using LiDAR sensors is proposed. This new approach is based on the variation of range measurements between neighbouring point clouds, using a two-step filter. The first filter examines the change in the standard deviation of neighbouring clouds. The second filter uses a change in distance and intensity between neighbouring pules to refine the results from the first filter and estimate the glass profile width before updating the cartesian coordinate and range measurement by the instrument. Test results demonstrate the detection and localisation of glass and the elimination of errors caused by glass in occupancy grid maps. This novel method detects frameless glass from a long range and does not depend on intensity peak with an accuracy of 96.2%.

## 1. Introduction

Mapping, navigation, and path planning have been one of the major research focus areas in robotics and auto industries in the past two decades [1,2,3,4,5,6,7]. Major contributions have been introduced, particularly to increasing the perception and understanding of the robots’ surrounding environment. Perception of the environment is greatly affected by the types of sensors used. Laser scanners are becoming common in many industries besides driverless vehicles, including rescue operations, medicine, robotics, and unmanned air vehicles. LiDAR (light detection and ranging) is the favoured laser sensor due to its high accuracy, wide and long scanning range, and high stability [8]. However, LiDAR is expensive and has major drawbacks when scanning in a transparent or specular reflective surface, such as glasses and mirrors. Hence, LiDAR sensors only account for diffuse objects [9]. However, most modern environments contain glass or other specular surface architectural features. Glass dividers, panned doors, and full-height windows are an example of such features [10].

Precision in localisation and mapping algorithms for robotics and driverless platforms is very critical. Using LiDAR sensors in a transparent environment causes the sensor to report inaccurate range data, leading to a potential collision triggered by errors in the map generated by the algorithm. Data collected by LiDAR sensors in an environment with a transparent and reflective object is also subject to a significant amount of noise caused by virtual cloud points created from the reflection of objects nearby the transparent materials [9]. Such reflected data points degrade the quality of the map. Thus, detecting glass and removing wrong range measures and point clouds generated by the reflection of objects (virtual points) in a LiDAR is critically important for laser-based grid mapping and the safety of autonomous robots.

**Figure 1:**
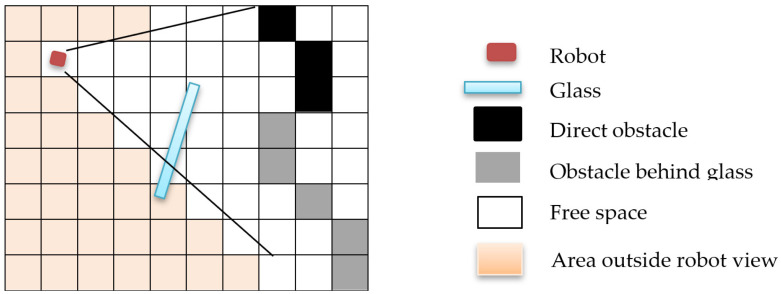
Illustration of a conventional occupancy map in a glass environment.

Occupancy grid mapping is a probabilistic method that maps the robot’s environment as an array of cells. Each cell holds the likelihood value that the cell is occupied. The basic assumption behind this method is that objects in the environment are detectable from any angle. Figure 1 illustrates how conventional grid mapping algorithms work in a glass environment. The orange square represents where the robot poses, and the blue rectangle represents the presence of glass in the environment. Obstacles that are directly hit by the LiDAR lasers are represented by black boxes, whereas the grey boxes represent obstacles behind the glass. All grid areas found where the glass is located should be mapped as occupied space to obtain an accurate representation of the environment and avoid potential collisions. However, traditional occupancy grid mapping fails to detect glass and identify its location.

In this paper, we propose a novel method that uses the variation of range measurements between neighbouring LiDAR point clouds to identify and localise the presence of glass in an indoor environment. This study quantifies the disparity between successive range measurements when the pulses pass through glass and hit objects and when the pulses hit objects with no presence of glass. We use two complementary filters to compute this variational difference. The first filter computes the rolling window standard deviation to locate the presence of glass. The second filter uses the output of the first filter as its input and combines measurements of distance and intensity to determine glass width profile and location. The method has been tested using an occupancy grid mapping algorithm to quantify and analyse its performance. As autonomous agents are becoming human assistants in indoor environments, which more likely contain a glass environment, our approach will have a great significant to identify and localise the presence of glass in such environments.

## 2. Related Work

Most of the existing literature addresses reflection detection in LiDAR and mapping glass using LiDAR intensity to try to overcome the challenges of reflection detection and glass detection separately. Other works use data fusion to overcome the glass and reflection detection problem. These works suffer from major reliability, precision, and generality issues [10].

For clarity, we have organised the related literature into four categories. The first category is literature that use intensity-based methods. The second category is publications that use glass frame detection methods. The third category is those that use a data fusion approach. The last category presents other approaches different than the above mentioned.

### 2.1. Studies Using Intensity-Based Methods

Shina et al. [11] proposed a glass detection mapping using state-based navigation decision and the reflection character of glass. Wang et al. [12] used a specular intensity profile around the normal incident angle to the glass panel surface and compared results with other transparent and reflective materials. Then, these results were finally integrated with a mapping algorithm. Jiang et al. [13] proposed a neural network algorithm that uses reflectivity, incident angle, and distance measurements to classify glass and non-glass objects using a laser range finder. Awais [14] and Foster [10] modelled the probability of receiving reflection back from the glass as a function of distance and an angle. [14] assumed this function as a Gaussian and identical for all glass, which leads to a gross error detection of glass. [10] used intensity peaks using similar assumptions. Kim et al. [15] used the reflective characteristics of a laser beam to classify diffuse, specular, and 1st and 2nd laser beam penetration. Results using this method show that the probability of occupied glass wall in a cell is lower than opaque, which has a direct impact on the quality of the map.

All intensity-based methods suffer from low accuracy when the distance or incidence angle of the laser beam increases [9,10]. Additionally, intensity peaks on a glass surface are only detectable where the LiDAR laser beams are perpendicular to the glass surface. This may be affected by the vibrations of the robot and elevation of the ground, consequently decreasing the reliability of intensity-peak based methods. Intensity is also greatly affected by the reflective properties of objects that the laser beams hit after specular reflection from the glass surface. Consequently, solely intensity-based glass wall detection is difficult, because there are a lot of factors affecting intensity.

### 2.2. Studies Using Glass Frame Detection Methods

Wang et al. [16] attempted to detect a window using LiDAR while driving in an outdoor setting. Wang initially clustered the cloud points and then tried to detect the facades of buildings. The surface normal was calculated using Principal Component Analysis (PCA) to detect potential cloud points representing a window. Pu et al. [17] also attempted to detect glass by using a reconstruction of building facade models from terrestrial laser scanners. However, both glass frame detection methods will only be applicable where a glass frame is present. Hence, this method fails in environments containing frameless glass.

### 2.3. Studies Using Data Fusion Approaches

Singh et al. [18] proposed a Bayesian filter approach to fusion a laser scanner and sonar to reduce mapping uncertainty caused by transparent objects. This approach was affected by the short-range measurement reading from the sonar sensor. Yang et al. [19] and Nagla et al. [20] also proposed a similar laser and sonar sensors fusion approach. The accuracy of [19] in transparent objects is poor, and the algorithm used by [20] is tested in a small experimental area. Data fusion approaches require additional sensors. Depending on the type of sensor, data fusion approaches may lead to high computational and financial cost.

### 2.4. Other Approaches

Singh et al. [21] proposed a concave shape sonar ring of multiple sonar sensors to reduce the uncertainty in sonar-based occupancy grid mapping due to specular reflection. This method only accounts for specular reflective objects. Yang et al. [22] used mirror symmetry in 2D LiDAR to mitigate mirror reflection. Yang applies a Gaussian model to predict the presence of a mirror following a Euclidean distance function to verify the presence of the mirror. While this approach only works for mirror-like reflective objects, it fails to detect transparent objects such as glass. Singh et al. [23] presented an error analysis method in laser scanners due to varying scanning angles with respect to surface refractivity or reflectivity and optical axis index of the target using a tilt mounting system. The author stated that tilting of the laser scanner generates errors in sensory information. Meng et al. [24] proposed an improved ray-casting Monte-Carlo localisation method to reduce scan matching error in a transparent surface environment.

Most of the existing work relies on specular reflection to detect glass, mirror, or shiny metal surfaces. However, the strength of a reflection varies among these different surfaces. Hence, the accuracy of these algorithms is higher in mirror and shiny metal surfaces than in glass surfaces. Therefore, in this study, we propose a method to detect and localise glass to improve occupancy map quality using LiDAR sensors.

## 3. Methods

**Figure 2:**
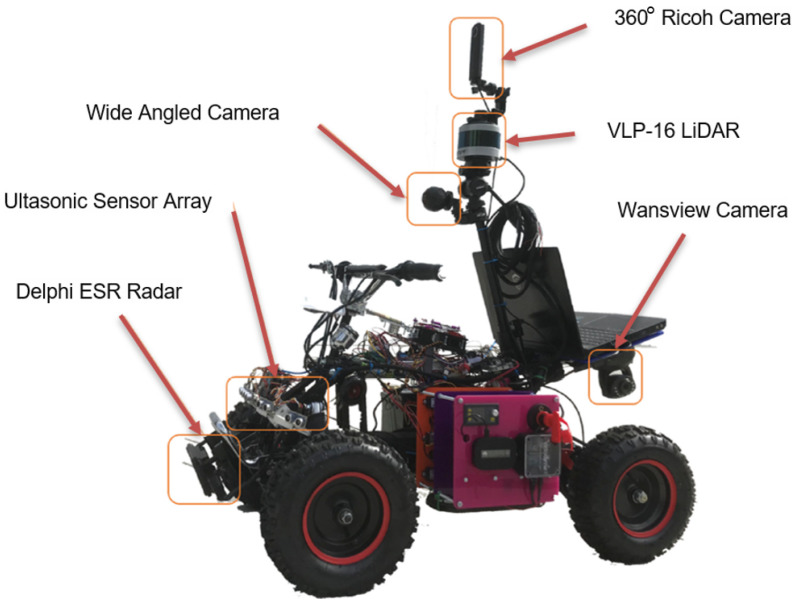
Loughborough University London Autonomous Testbed used for data collection.

Initially, we collected data using the Loughborough University London Autonomous Testbed Figure 2. The testbed has six different sensors installed: three cameras 360° Ricoh, Wansview, and wide-angle camera to collect visual data; an ultrasonic sensor to collect near-distance range measurements; and a Delphi ESR Radar sensor to measure the front side far-distance range measurement and speed data. The LiDAR data used in this study, which is generated from a Velodyne VLP-16 LiDAR apparatus, was collected while the car was driven autonomously by leveraging data from the sensors mounted on the testbed. This sensor has a maximum range of 100 m with 16-channels, taking a total of 300,000 measurements per second. This device captures 360° and 30° on the horizontal and vertical axis, respectively. The data are collected in different types of glass and glass installations, including single and double-glazed structures.

**Figure 3:**
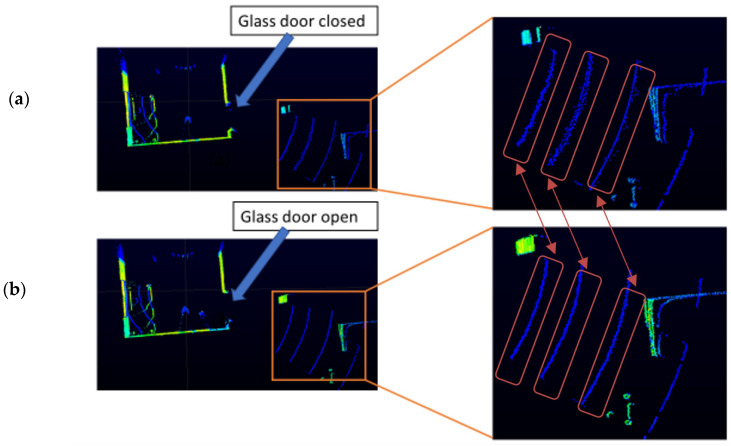
Displays image of LiDAR (light detection and ranging) distance measurements that are color coded by intensity (i.e., calibrated reflectivity). These measurements are collected in a room containing a glass door. (**a**) is collected while the glass door a is closed, and (**b**) is collected while the glass door is open. Both of these data are collected whilst the robot is at the same origin. The area detected behind the glass door is displayed in the orange box and the zoomed image of the red box is displayed in the right side of the image. As shown in the zoomed part of (**a**,**b**), the consecutive laser pluses distance measurement does not follow a similar pattern when the glass door is opened and closed. Looking closely, the three red boxes in (**a**,**b**) show the variation of distance measurement of neighbouring point clouds. The distance measurement of neighbouring lasers that passes through glass and hits the floor (when the glass door is closed) shows a relatively higher variation in the range measurement than when the point clouds when the glass door is open.

The range measurements in the orange box in Figure 3a versus Figure 3b have a slight difference. Although the difference is very small, it is mathematically significant. The difference in range measurements is smaller or larger depending on the height of the LiDAR from the ground and the angle of elevation of the LiDAR. The VLP-16 LiDAR sensor used in this study has an array of 16 infrared lasers, and each laser fires approximately 18,000 times per second. Each of the laser’s 16 channels are fixed at a certain elevation angle in relation to the horizontal plane of the LiDAR apparatus. Each of the 16 infrared lasers are assigned a specific laser ID number from 0 to 15 counting from the bottom to the upper channel. In this experiment, the LiDAR is mounted at 90 cm from the ground, and range difference between neighbouring pulses is largest in laser IDs 4, 6, and 8 with vertical angles −110, −90, and −70, respectively. When laser pulses pass through a glass medium, the difference in range measurement of neighbouring point clouds is higher than when lasers hit an object directly.

We conduct the experiment frame by frame because we aim to achieve our objective accurately starting from the very first scan. The experimental setup is designed in such a way to makes the proposed algorithm robust by pressing it to produce results from only one a single scan. Therefore, our algorithm does not depend on consecutive scan matching which leads to a potential gross error over time. Our experiment is set at 5 frames per second. Figure 3a,b shows a scan of a single frame from the LiDAR.

**Figure 4:**
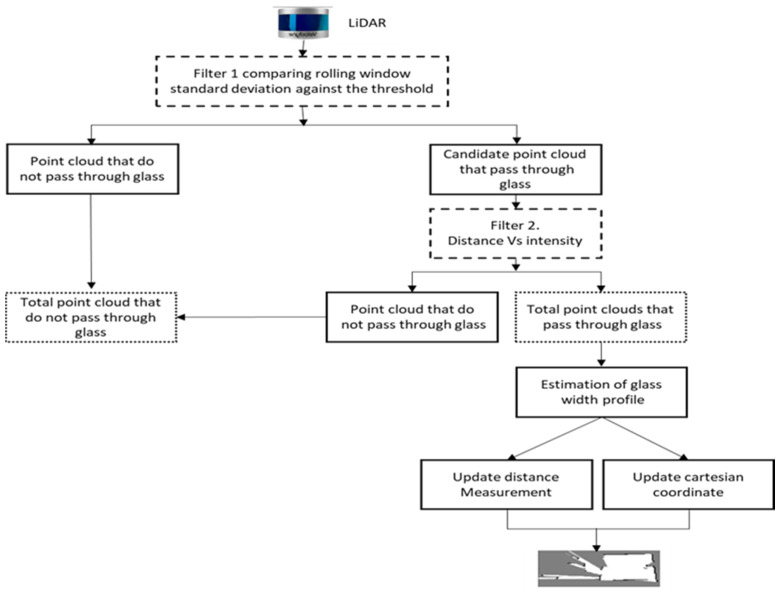
Proposed method layout.

Figure 4 classifies the point clouds in two groups by quantifying the range difference. First, we manually extracted a set of point clouds that pass through glass and classify them as PCoG (Point Cloud ofGlass). We follow the same procedure and manually extract the point clouds, which directly hit objects and classify them as PCoO (Point Clouds of Object). Then, we calculate the standard deviation of range measurements between the two groups. σ represents the standard deviation, μ is the mean, and N is the total number of point clouds included in each group.

(1) $$\sigma PCoG=\;\sqrt{\frac{\sum^{\;}{\left(PCoG-\mu \right)}^{2}}{N}}$$

(2) $$\sigma PCoO=\;\sqrt{\frac{\sum^{\;}{\left(PCoO-\mu \right)}^{2}}{N}\;\;\;\;\;}$$

We apply Equations (1) and (2) in several point cloud datasets from single- and double-glazed glass environments. Then, we compute the threshold (Trh) between the two groups as follows:

(3) $$Trh=\;\;\frac{1}{2}\;\sum^{\;}\sigma PCoG+\sigma PCoO$$

The result of Equation (3) is used as a threshold to compare against the new neighbouring data inputs from the LiDAR apparatus. We did not compute the standard deviation of two consecutive laser pulses to compare against the threshold, as the standard deviation different between σPCoG and σPCoO will be very small. Instead, we use a sliding window to compute the standard deviation of a group of neighbouring incoming pulses, as this simply widens up the gap between standard deviation of σPCoG and σPCoO. The mathematical representation of the sliding window size ($W_{i}^{H,J}$) used in this study is declared in Equation (4). H and J are the number of samples included before and after the point cloud ($x_{i\;}).$ In other words, H is the upper limit of the sliding window for sample ($x_{i\;})\;$and J is the lower limit of the sliding window for the same sample ($x_{i\;})$. We ran several experiments to decide the sliding window size and found size 11 to have the highest accuracy. Hence, we use 5 samples before and after sample$\;\left(x_{i\;}\right).$ In other words, both H and J=5.

(4) $$\;\;\;\;\;\;\;\;\;W_{i}^{H,J}=\left\{x_{i-H,\;\;\;.\;\;.\;\;.\;\;,\;}x_{i,\;\;\;.\;\;\;.\;\;\;.\;\;,\;}x_{i+J\;}\right\}\;\;\;\;\;\;\;\;\;\;\;\;\;\;\;\;\;\;\;\;$$

Using the window stated in Equation (4), we calculate the standard deviation of the range measurement in each window. $\sigma_{Wi}$ represents the standard deviation of each window. Lr represents the LiDAR range measurement. $\mu_{i}\;$is the mean of the laser measurements inside the window.

(5) $$\sigma_{wi}=\frac{1}{\left(\left|W_{i}^{H,J}\right|\right)}\;\;\;\underset{LR\;\in \;W_{i}^{H,J}\;}{\sum }{\left(\;Lr-\;\mu_{i}\right)}^{2}$$

We subsequently apply Equation (5) to classify each window as a PCoO or as a candidate for PCoG by comparing the result with the threshold (first filter), as presented in Figure 4.

Equations (1)–(3) are performed on hand-labelled PCoG and PCoO data. In filter 1, we primarily use the standard deviation difference between the two groups to classify incoming point clouds as PCoG and PCoO. Hence, we use the average of the hand-labelled data’s standard deviation (Equation (3)) as a threshold. Therefore, every 11 new incoming point cloud data are assigned to group (the sliding window size (Equation (4))) and the standard deviation of each of each window (Equation (5)) is compared against Equation (3). If the result is above the average, then these point clouds are labelled as potential PCoG, and if the result is below the threshold, it is classified as PCoO. The pseudocode of Algorithm 1 is presented below.
**Algorithm 1.** Filter 1**Input** *W_i_^H,J^***←** rolling window *P*
**←** the scan point clouds *Trh*
**←** threshold **for** each point clouds run a rolling window **do**           Calculate the *STDV of window*              **If**
*STDV of the window*
**>** threshold **then**                            Store the point cloud to potential ***PCoG***              **else**                            store the point as ***PCoO***              **end if** **end**

**Figure 5:**
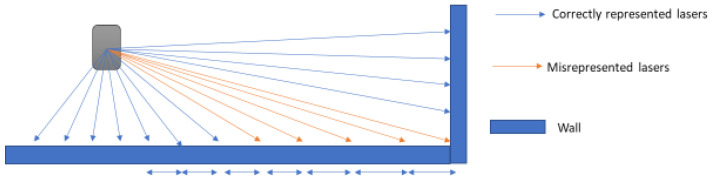
Laser beam hitting a straight wall. The difference between each neighbouring blue pules range measurement is smaller, and the difference in the orange pulses is larger.

When lasers hit a straight object such as a wall, range measurements between neighbouring pulses either increase or decrease depending on the rotation direction of the apparatus, as illustrated in Figure 5.

**Figure 6:**
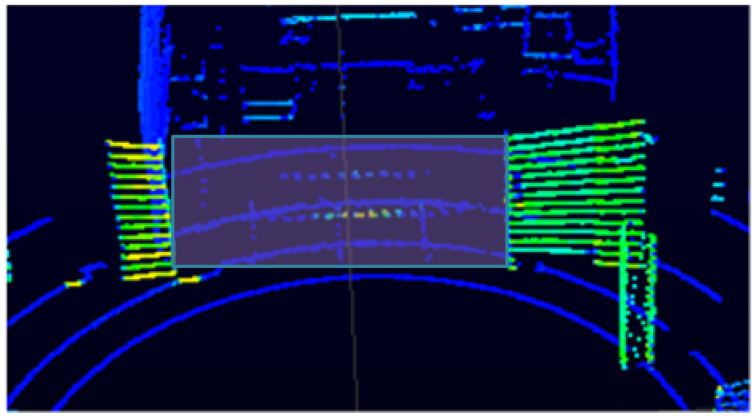
Intensity map from LiDAR (light detection and ranging) sensor. Intensity lowers when distance increases (in other words, the intensity lowers when a laser beam passes through the glass due to the increase in distance).

This characteristic has a significant impact on the standard deviation, as it tends to increase its value, which may lead to the wrong assignment of some point clouds hitting the straight surface as a PCoG. Hence, we run a second filter on all points clouds assigned as PCoG by filter one. The second filter is designed based on the following beliefs:

- Distance increases and intensity decreases at the first point when a LiDAR pulse passes through the glass as it is expressed in (Figure 6), and
- Distance decreases and intensity increases at the last point when a LiDAR pulse passes through the glass (Figure 6).

All point clouds that do not meet the second filter criteria are assigned as PCoO and added together with filter one PCoO to get the total, as illustrated in Figure 4. We also use the second filter (Algorithm 2) to confirm point clouds assigned as PCoG are in the correct glass width profile (within the interval of intensity fall and rise (Figure 6)).
**Algorithm 2**. Filter 2**Input** ***PCoG* ← **Potential glass hitting point clouds filtered by algorithm one ***PCoO* ← **Non-glass object hitting point clouds by algorithm one ***I* ← **Intensity ***Dis* ← **LiDAR range measurement **for** all point clouds except for those filtered as ***PCoO*** by algorithm one **do**
              **if *Dis*** increases at the first point
Where a pulse passes through glass
                            **AND**
                             **if**
***I*** decreases at the same point, **then**
                              Store this point as beginning of window profile width
                            **end if**
              **end if**
              **if *Dis*** decreases at the end point
Where a pulse passes through glass
                            **AND**
                             **if *I*** increases at the same point **then**
                              Store this point as end of window profile width
                            **end if**
              **end if**
**end**
**for** all PCoO **DO**
                      **if** between the beginning and end
                      profile width **then**
                            accept as a ***PCoO***
                      **else**
                            add the point clouds to ***PCoO***
                      **end if**
**end**


**Figure 7:**
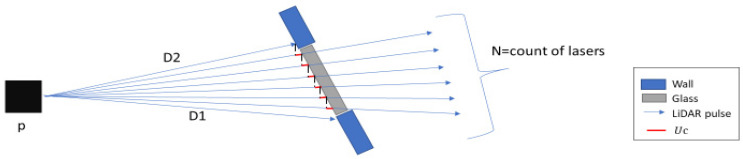
Illustration of how cartesian coordinates and range measurement is updated.

Finally, we update the cartesian coordinates and the range measurement of all the point clouds assigned as PCoG by the second filter. Let $D_{2\;}\;\mathrm{and}\;D_{1}$ represent the range and cartesian coordinates measure at the end and beginning of a glass region respectively (i.e., the beginning and the end measurement of consecutive point clouds assigned as PCoG by the second filter, illustrated in Figure 7). Uc is the value-added on each consecutive point. N is the total count of consecutive lasers.

(6) $$\;Uc\;=\frac{\;D_{2}\;-D_{1}}{N}$$

Once the glass width profile is known from Algorithm 2, then, we extract the upper and lower limit of the glass width profile’s distance measurement (D1 and D2), the cartesian coordinate at the beginning and end of the glass width profile (X1 and X2) and (Y1 and Y2) together with the total number of laser beams within the upper and the lower limit interval. We use these as the input for algorithm three to update distance and cartesian coordinates. Algorithm 3, presented below, is for updating distance measurement. Cartesian coordinates can be similarly updated by changing D1 and D2 by (X1 and X2) and (Y1 and Y2), respectively.
**Algorithm 3.** Update distance (Similar method used for updating cartesian coordinates) **Input**           ***N* ← **Count of laser           ***D1* ← **Distance measurement of the end of the glass frame           ***D2* ← **Distance measurement of the beginning of the glass frame **for** each point clouds to the length of ***N* do**                            $\frac{D2-D1}{n}$                     Store the result to **Uc**                     Add **Uc** to the range measurement                     Store the result
**end**


## 4. Experiments and Results

This section consists of three experiments conducted to demonstrate the usability of the proposed algorithm. These experiments use single- and double-glazed glass. All the glass used in these experiments are glass walls. In the rooms where experiments 1 and 2 were conducted, one wall was partially glass. In the room where experiment 3 was conducted, one wall consisted of two separate glass panes.

**Figure 8:**
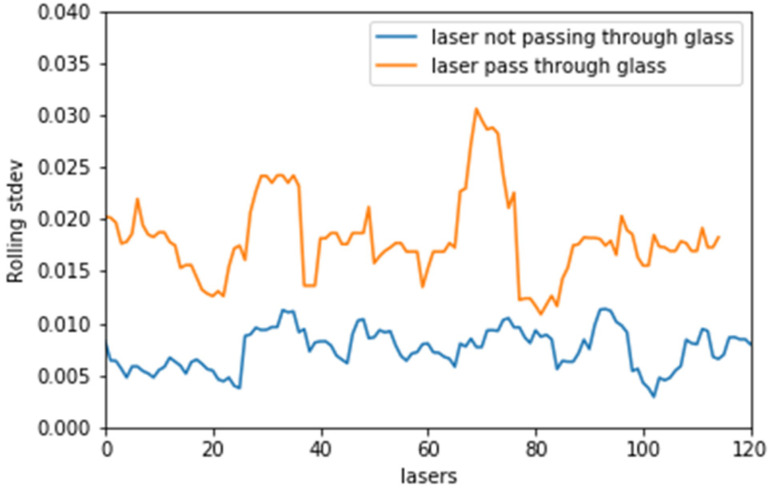
Rolling standard deviation (calculated using rolling window i.e., the standard devation of each window) of the laser range between point clouds passing through glass and point clouds directly hitting an object.

Initially, we present the finding that in all the experiments, there is a marginal difference in the rolling window standard deviation (rolling stdev) of the laser range measurements between point clouds that pass-through glass and those that do not. Figure 8 illustrates this margin.

The experiments presented hereafter have two primary aims: firstly, to identify and locate the presence of glass in the three experimental setups and, secondly, if there is a presence of glass, to update the lasers pules distance by recalculating the distance between the LiDAR apparatus and the identified glass.

### 4.1. Experiment 1: Office-Like Environment

**Figure 9:**
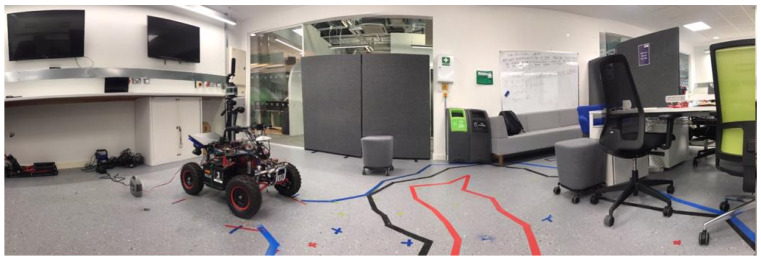
Experiment 1 room.

This experiment room is conducted in an office-like room. The room has a glass wall located at the corner of the left side, as seen in Figure 9. The glass wall in experiment 1 is a single glazed glass. The height of the glass is 2.5 m, and the width is 1.5 m. Point clouds behind the glass wall are unwanted, since they represent objects beyond the glass. There was a 3-m distance between the glass wall and the LiDAR apparatus.

Using the proposed method, the point clouds that pass through glass are identified using Equation (3). The width of the glass is identified using the second filter of the proposed algorithm. Then, the distance and cartesian coordinates of the point clouds that are within the range of the identified glass are updated using Equation (4).

**Figure 10:**
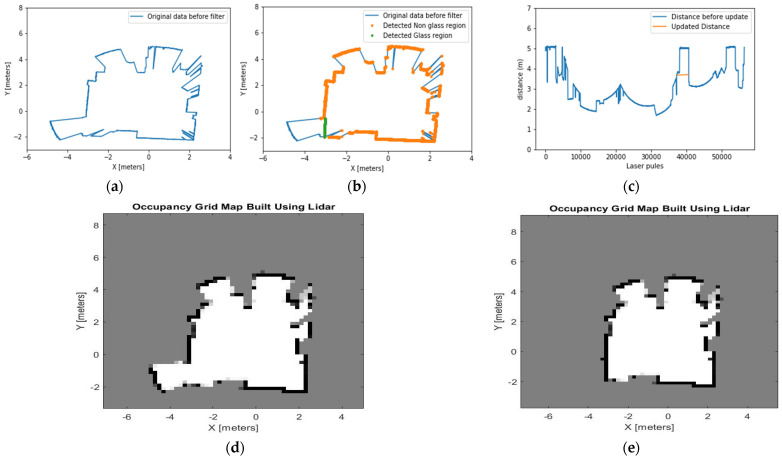
(**a**) A map built by the original LiDAR data shows no recognition of the presence of glass in the office, (**b**) the glass region is detected and presented in a green dotted line, (**c**) the distance measurement is updated based on the detected glass region and presented in the orange line, (**d**) the occupancy grid map built by using the original LiDAR data before the glass region is detected. This map shows the area beyond the glass region as a free space, (**e**) occupancy grid map built using the proposed method recognises the areas beyond the glass as occupied spaces.

All maps displayed in the results sections are 2D maps presented on an XY plane. The result in green (Figure 10b) shows the updated cartesian coordinates of the identified glass regions. In Figure 10c, the orange line shows the updated distance measurement. Figure 10d,e show the comparison of the traditional grid map and grid map constructed using the proposed method. The traditional method wrongly identifies the glass region and points beyond the glass region as free space. The proposed method correctly identifies the LiDAR point clouds that pass through the glass wall. This algorithm also updates the distance and cartesian coordinates to approximate the location of the glass. Finally, point clouds beyond the glass region are labelled as occupied space.

### 4.2. Experiment 2: Room with a Corridor in Front

**Figure 11:**
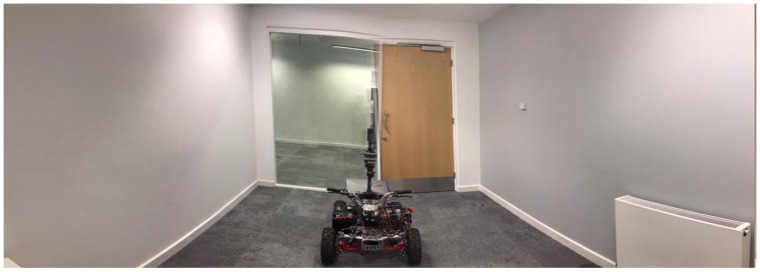
Experiment 2 room.

The second experiment is conducted in an empty room with a double-glazed glass wall located on the front side of the room as shown in Figure 11. There was a 2-m distance between the LiDAR apparatus and glass wall. The height of the glass wall is 2.20 m and the width is 1.5 m.

**Figure 12:**
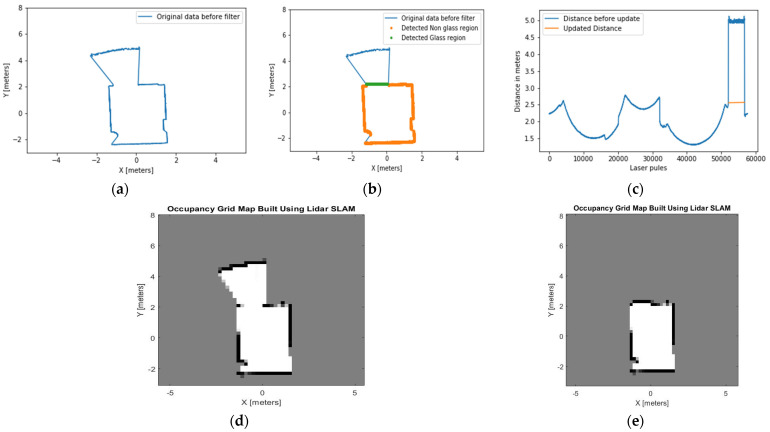
(**a**) A map built by the original LiDAR data shows no recognition of the presence of glass in the empty room, (**b**) the glass region is detected and presented in a green dotted line, (**c**) the distance measurement is updated based on the detected glass region and presented in the orange line, (**d**) the occupancy grid map built by using the original LiDAR data before the glass region is detected. This map shows area beyond the glass region as a free space, (**e**) the occupancy grid map built using the proposed method recognises the area beyond the glass as occupied spaces.

Our algorithm filters the point clouds that pass through glass and identifies the width of the glass boundary using Equation (5) and filter two, respectively. The conventional occupancy grid map (Figure 12d) assumes there is a free space pathway between the room and the corridor. Figure 12e presents the map produced by our method. This approach effectively identifies the glass area and estimates the glass width. It produces a correct representation of the environment by updating the cartesian coordinates and the distance measurement of the LiDAR.

### 4.3. Experiment 3: Room with Two Glass Walls

**Figure 13:**
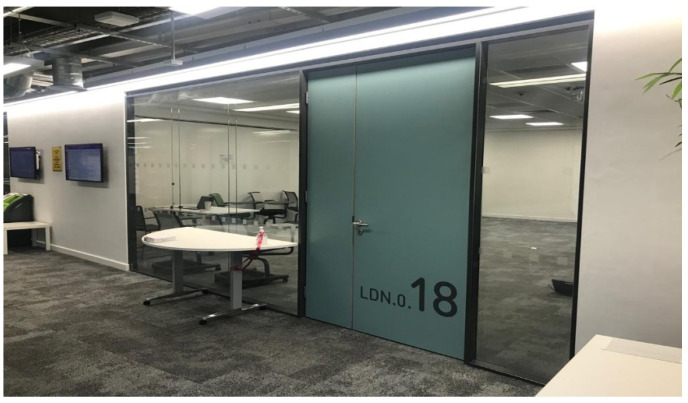
Experiment 2 room.

The third experiment is conducted in a room that has two single glaze glass walls, as shown in Figure 13. The first glass was put in a 2-m distance from the LiDAR apparatus and had an area of 0.75 m × 2.20 m. The far side of the second glass has a 4-m distance from the LiDAR apparatus and has an area of 2.5 m × 2.20 m. A 1-m wide wall separates the two glass planes.

**Figure 14:**
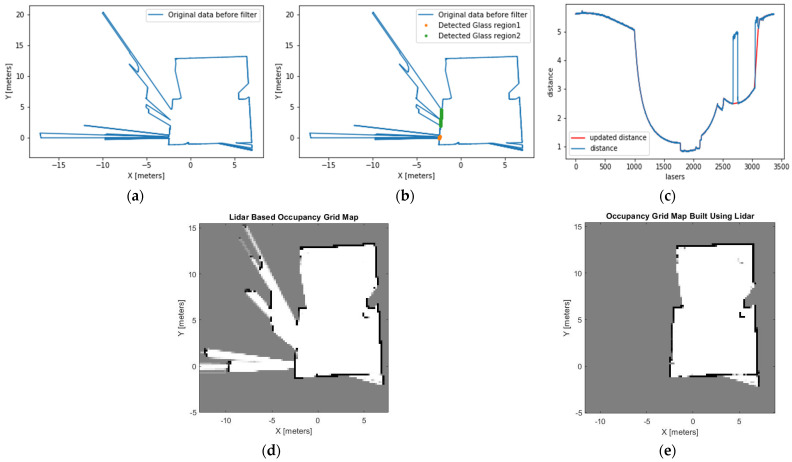
(**a**) A map built by the original LiDAR data shows no recognition of the presence of both glass in the room, (**b**) lasers that pass through the glass are identified and marked by the orange color, (**c**) the distance measurement is updated based on the two detected glass regions and presented in the red line, (**d**) the occupancy grid map built by using the original LiDAR data before the glass region is detected. This map shows the area beyond the glass region as a free space, (**e**) occupancy grid map built using the proposed method recognises the area beyond the glass as occupied spaces.

Both glass walls in this experiment are identified and located. The identified width of the glass in this experiment is a little greater than the dimension of the glass because we applied a higher window size to filter the point clouds. As a result, non-glass walls near the glass end and start points are labelled as a glass. Nevertheless, this does not affect the map produced (Figure 14e), as the purpose of the algorithm is to identify glass and to assign the glass as occupied space.

Using the proposed method, in all three experiments, glass walls are displayed as occupied regions on the respective occupancy grid map cell.

**Figure 15:**
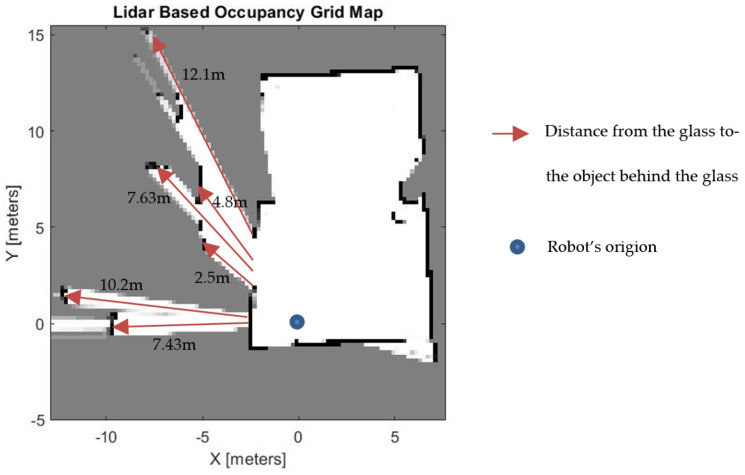
Evaluation of different distances between the glass and objects behind the glass.

We conducted experiment 3 in an environment where objects, which are behind the glass, are placed at different distances from the location of the glass. Such an environment makes it viable to see the performance of the proposed algorithm with respect to the distance of objects behind the glass. While the closest object to the glass is placed 2.5 m away, the farthest is located 12.1 m from the glass. Figure 15 shows that our algorithm effectively works despite the difference in distance of objects behind the glass. However, theoretically, it will be difficult for our algorithm to detect glass if an object is placed right behind the glass. This is due to the overlap between *PCoO*’s and *PCoG’s* rolling standard deviation. However, since we are proposing this algorithm to improve occupancy gride mapping, such case has no effect. If objects are placed right behind the glass, then we can assume that there will be no free space to be detected between the object and the glass.

The quantitative performance evaluation of the proposed algorithm is challenging since it is hard to obtain a ground truth point cloud with glass areas detected [25]. Hence, we compare the proposed method result with ground truth data generated by hand labelling each point cloud as Point clouds passes through glass and Point clouds does not pass through glass. The method’s accuracy is quantified based on methods by Zhao et al. [9] and Foster et al. [10]. Point clouds passing through glass were considered accurate if the point cloud is in the subset of hand-labelled Point clouds passes through glass. If the point cloud is not in the subset, it will counted as false positive. Table 1 shows the experiment results compared to the hand-labelled ground truth. The mean accuracy of glass correctly detected in the proposed method is 96.2%.

We also evaluate the proposed method’s accuracy of the updated distance. A sticker was attached to selected places on the glass during data collection. The ground truth for the true position of the glass (the true distance from the robot’s pose to the glass) is collected by using these stickers attached to the glass. The cloud points that hit the sticker are extracted, and their respective range measurement is used as the ground truth distance. Then, the updated distance results from the proposed algorithm are compared against the ground truth distance. Then, we calculated the Root Mean Square Deviation (RMSD) and compared against the approach of Zhao et al. [9]. As shown in Table 2, this method gives a better estimation of the glass location.

## 5. Conclusions

This paper presents a novel methodology to perform occupancy grid mapping in the presence of glass. Existing reflection detection algorithms perform poorly in the presence of glass. Therefore, in this paper, we focus on glass detection by itself.

The proposed method is based on the variation of neighbouring LiDAR point clouds when the LiDAR pulses pass through glass. Results show that there is a variation of neighbouring pulses’ distance measurement when the pulses pass through glass versus when the pulses directly hit objects. We classify LiDAR pulses into two groups: those that pass through glass and those that directly hit objects. Then, we apply two filters using intensity and range discrepancy to identify the boundary of the glass. Finally, we update the cartesian coordinates and the distance measurement of the LiDAR. Then, we show the usability of this method using occupancy grid maps that demonstrate improved map quality with a single scan. This approach effectively identifies and localises glass and improves indoor mapping quality using LiDAR sensors.

The key findings and contributions of this paper can be summarised as follows:

- LiDAR range measurements exhibit a different character when the pulses pass through glass
- We proposed a novel method to detect and localise glass using LiDAR
- We proposed a new approach to update distance and cartesian coordinates measurement from a LiDAR apparatus to compensate the incorrect reading due to the presence of glass.
- Our approach effectively improves traditional occupancy grid mapping by eliminating a false positive free space.

In the future, this approach can be integrated with a camera sensor to investigate the algorithms robustness for indoor and outdoor dynamic environment in real time.

## Figures and Tables

**Table 1:** Result of glass detection.

	Ground Truth Point Clouds Passes through Glass	Ground Truth Point Clouds Does Not Pass through Glass	Our Method Point Clouds Passes through Glass	Ground Truth Point Clouds Does Not Pass through Glass
Experiment 1	2823	56,925	96.3%	95.6%
Experiment 2	2774	58,064	96.5%	92.3%
Experiment 3	4901	53,746	95.9%	94.2%
Proposed method glass correctly detected (avg)				96.2%
VisAGGE [10] method glass correctly detected				94.90%

**Table 2:** Accuracy of glass location.

	RMSD
Experiment 1	0.0180
Experiment 2	0.0814
Experiment 3	0.0291
Proposed method (Avg)	0.0429
Zhao et al. approach [9] (Avg)	0.0500

## Data Availability

Not applicable.
